# Women Versus Men with Chronic Atrial Fibrillation: Insights from the Standard Versus Atrial Fibrillation spEcific managemenT studY (SAFETY)

**DOI:** 10.1371/journal.pone.0065795

**Published:** 2013-05-29

**Authors:** Jocasta Ball, Melinda J. Carrington, Kathryn A. Wood, Simon Stewart, the SAFETY Investigators

**Affiliations:** 1 Centre of Research Excellence to Reduce Inequality in Heart Disease, Preventative Health, Baker IDI Heart and Diabetes Institute, Melbourne, Australia; 2 Department of Epidemiology and Preventative Medicine, Monash University, Melbourne, Australia; 3 School of Nursing, Duke University, Durham, North Carolina, United States of America; Loyola University Chicago, United States of America

## Abstract

**Background:**

Gender-based clinical differences are increasingly being identified as having significant influence on the outcomes of patients with cardiovascular disease (CVD), including atrial fibrillation (AF).

**Objective:**

To perform detailed clinical phenotyping on a cohort of hospitalised patients with chronic forms of AF to understand if gender-based differences exist in the clinical presentation, thrombo-embolic risk and therapeutic management of high risk patients hospitalised with chronic AF.

**Methods:**

We are undertaking the Standard versus Atrial Fibrillation spEcific managemenT studY (SAFETY) - a multi-centre, randomised controlled trial of an AF-specific management intervention versus usual care. Extensive baseline profiling of recruited patients was undertaken to identify gender-specific differences for risk delineation.

**Results:**

We screened 2,438 patients with AF and recruited 335 into SAFETY. Of these, 48.1% were women who were, on average, 5 years older than their male counterparts. Women and men displayed divergent antecedent profiles, with women having a higher thrombo-embolic risk but being prescribed similar treatment regimens. More women than men presented to hospital with co-morbid thyroid dysfunction, depression, renal impairment and obesity. In contrast, more men presented with coronary artery disease (CAD) and/or chronic obstructive pulmonary disease (COPD). Even when data was age-adjusted, women were more likely to live alone (odds ratio [OR] 2.33; 95% confidence interval [CI] 1.47 to 3.69), have non-tertiary education (OR 2.69; 95% CI 1.61 to 4.48) and be symptomatic (OR 1.93; 95% CI 1.06 to 3.52).

**Conclusion:**

Health care providers should be cognisant of gender-specific differences in an attempt to individualise and, hence, optimise the management of patients with chronic AF and reduce potential morbidity and mortality.

## Introduction

Combating cardiovascular disease (CVD) in women, in all its forms, represents an ongoing challenge [1] – from acknowledging its wider impact from a population burden perspective (it remains the single largest cause of death in women [1]) to formulating a robust evidence-base for those affected. In both low-to-middle [2], [3] and high-income countries [3], the natural history of CVD and age profile of affected women are typically different from their male counterparts. This requires careful interpretation of key clinical trials of new treatment strategies where women are almost always in the minority [4], [5]. Underlying gender-based differences in the pattern and outcomes of CVD presentations are complex; ranging from intrinsic drivers such as anatomic, physiological and genetic factors, to differences in health behaviours, delays in recognising and responding to symptoms, to critical health care factors such as under-utilisation of gold-standard diagnostic tests and treatments [6]–[8]. The current description of “typical” cardiac symptoms is based primarily on white, middle-aged men and has contributed to misconceptions in clinicians and lay individuals, leading to inaccurate diagnosis and a delay in women seeking treatment [9].

The conundrum of ensuring gender-based equity in outcomes is becoming increasingly evident in relation to atrial fibrillation (AF). Predicted to be the next CVD-related epidemic due to the progressive ageing of the population and successful treatment of many of its antecedents, it is the most common cardiac arrhythmia observed in clinical practice and accounts for approximately one third of hospitalisations for all arrhythmias [10]. With a current overall population prevalence of 2% to 3%, age-adjusted prevalence rates are higher in males than females [10]. However, the absolute number of women with AF exceeds that of men due to their greater longevity [11], [12]. Indeed, after 75 years of age (the median age of AF diagnosis) approximately 60% of individuals with AF are women [13]. Known clinically relevant differences between women and men with AF currently relate only to the prevalence, presentation and outcomes [14]–[17]. However, fundamental biological differences between women and men relating to the underlying pathogenesis of AF have only just begun to be elucidated.

### Study aims

Consistent with the development of chronic heart failure (CHF) management programs to support the application of gold-standard therapeutics, with more equitable recruitment of women into key clinical trials of the same [4], we are applying a systematic approach to risk delineation and optimising management of hospitalised patients with chronic forms of AF. As such, we are undertaking the Standard versus Atrial Fibrillation spEcific managemenT studY (SAFETY) [18]. We prospectively hypothesised that gender-based differences would exist in the clinical presentation, thrombo-embolic risk and therapeutic management within the cohort of high risk patients hospitalised with a diagnosis of chronic AF screened for and subsequently recruited into SAFETY. We further hypothesised that subject to detailed clinical phenotyping, these differences would have important clinical implications in respect to disease management requirements.

## Methods

### Ethics statement

Ethics approval was obtained from the Central Northern Adelaide Health Service Ethics of Human Research Committee, Metro South Health Service District Human Research Ethics Committee, Melbourne Health Human Research Ethics Committee, Western Health Office for Research and the ACT Health Directorate Human Research Ethics Committee. Written informed consent was obtained from each study participant prior to study procedures being conducted.

### Study setting

The overall purpose and design of SAFETY has been described in greater detail previously [18]. In brief, 335 hospital in-patients with chronic forms of AF were recruited from three tertiary hospitals in Australia. All were subject to comprehensive baseline profiling. Patients were randomised to either usual post-discharge care or a home-based, multidisciplinary, AF-specific intervention designed to reduce morbidity and mortality.

### Study participants

A systematic screening program to identify eligible inpatients was conducted at each participating hospital. Patients were approached for recruitment if they were English-speaking, had a documented diagnosis of recurrent paroxysmal (i.e. recurrent episodes by history as documented on ECG), persistent or permanent AF; were living independently in the community or their own home post-hospitalisation; and were able and willing to provide written informed consent to participate. Patients were excluded if they were aged less than 45 years, had a primary diagnosis of valvular heart disease, were scheduled for catheter ablation of their AF, had pre-existing CHF as evidenced by the combination of symptoms indicative of NYHA Class III-IV with a documented left ventricular ejection fraction (LVEF) less than 45%, or had a transient form of AF. Patients with dementia, as assessed on routine evaluations conducted by hospital clinical teams, were also excluded as were patients who were too unwell at the time when testing was attempted.

## Data Collection

### Baseline profiling

Comprehensive profiling of each patient subsequently enrolled in SAFETY involved the collection of basic socio-demographic, past medical history and current admission data. In addition, questionnaires were administered at the time of recruitment (wherever feasible) to assess lifestyle (i.e. cigarette and alcohol consumption, exercise and sleep habits), health-related quality-of-life (HRQoL – via EuroQol 5-dimension scale [EQ-5D] [19] and Short Form 12 [SF-12] [20]), depression status (via the ARROLL [21] ± Centre for Epidemiological Studies Depression Scale [CES-D] [22]) and cognitive function (via the Montreal Cognitive Assessment [MoCA] [23]). Furthermore, a comprehensive profile of each patient’s experiences with AF was derived, including sub-type classification, patient-reported symptoms, patient-reported triggers, patient awareness (of AF episodes, symptoms and triggers), previous and current treatment plans. The CHA_2_DS_2_-VASc score was used as a risk stratification tool to assess requirements for antithrombotic therapy [24]. For the current research, the CHA_2_DS_2_-VASc score was scored from 0-8 (not 0–9 as is the norm) due to the exclusion of patients with chronic heart failure. All questionnaire data were collected prospectively during face-to-face semi-structured interview by trained personnel according to standardised criteria. Self-reported AF profile data was collected utilising an investigator designed interview guide that specifically asked about symptoms experienced and triggers associated with episodes.

### Statistical analyses

Where appropriate, descriptive values are presented as mean (± standard deviation [SD]) for continuous variables or a proportion for categorical variables. Differences between women and men were assessed using the chi-square (X^2^) test for dichotomous variables and independent t-test for normally distributed continuous variables. Age-adjusted comparisons of the socio-demographic, risk and clinical profile of patients were examined with a simple regression model. Only statistically significant odds ratios (ORs) are presented. Data were analysed using SPSS, V.20. A probability value of p<0.05 (two-sided) was considered statistically significant.

## Results

### Patient screening and recruitment

**Figure 1:**
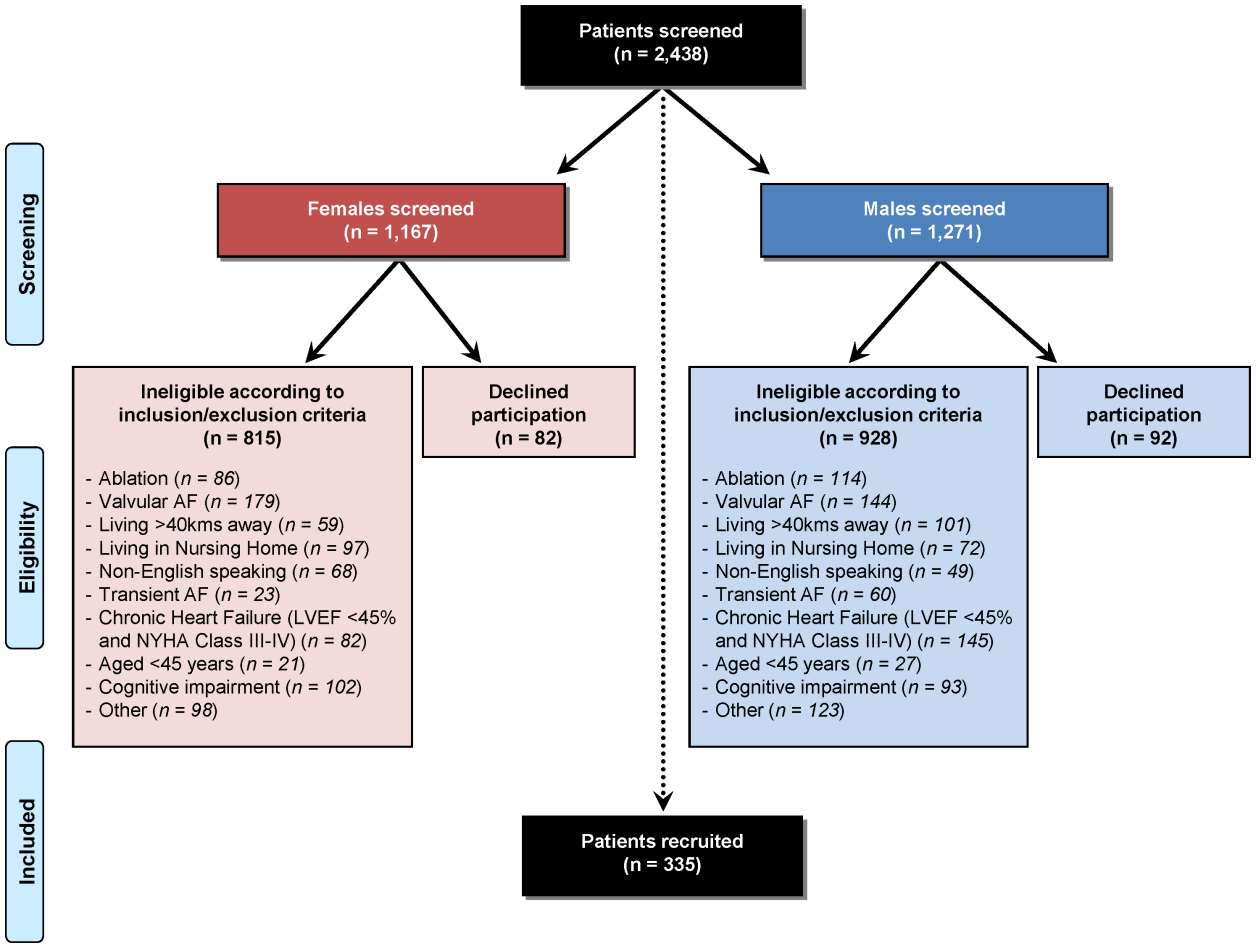
Flow chart of SAFETY recruitment stratified by gender.

**Figure 1** shows the profile of screened and recruited patients according to gender. Overall, of the 2,438 patients with AF (as a primary or secondary diagnosis) screened for entry into SAFETY across the three hospital study sites, n** = **335 (13.7%) were enrolled. Of these, 1,167 (47.9%) were women and 1,271 (52.1%) were men. The mean age of these patients who presented to hospital with AF was 75 years ±13 years. Women presented, on average, 4 years older than men (77 years ±13 years versus 73 years ±13 years, respectively).

### Socio-demographic profile

As shown in **Table 1**, 161 (48.1%) recruited patients were women aged, on average, 5 years older than their male counterparts. Over half of these women were aged 75 years or more compared to just over a third of men in the same age group. In addition, more than half of female patients were currently living alone (as a result of being divorced/separated [4.8%], having been widowed [17.6%] or never being married [2.1%]; p<0.001). Further, the proportion of women who had formal education at a tertiary level was less than half that of male participants.

**Table 1:** Baseline characteristics of females and males hospitalised with chronic AF.

	Females(n = 161)	Males(n = 174)	p value
**Socio-demographic profile**			
Mean age (years)	74.0±10.3	69.3±11.6	**p<0.001**
≥75 years	81 (50.3%)	64 (36.8%)	**p = 0.013**
Living alone (%)	82 (50.9%)	50 (28.7%)	**p<0.001**
Tertiary level education obtained (%)*	30 (19.0%)	68 (40.7%)	**p<0.001**
Employed in unskilled/semi-skilled occupation (%)^†^	89 (71.8%)	73 (48.0%)	**p<0.001**
**AF-specific profile**			
Paroxysmal AF diagnosis (%)	4 (2.5%)	9 (5.2%)	p** = **0.203
Persistent AF diagnosis (%)	140 (87.0%)	149 (85.6%)	p** = **0.725
Permanent AF diagnosis (%)	17 (10.6%)	16 (9.2%)	p** = **0.676
Rate Control (%)	102 (63.4%)	112 (64.4%)	p** = **0.847
Rhythm Control (%)	59 (36.6%)	62 (35.6%)	p** = **0.847
Mean CHA_2_DS_2_-VASc score	4±2	3±2	**p<0.001**
**Antecedents**			
Mean HR on admission (bpm)	101±34	97±33	p** = **0.268
Mean HR at discharge (bpm)	74±15	75±17	p** = **0.883
Mean BMI (kg/m^2^)^≠^	30.5±7.9	28.8±5.3	**p = 0.033**
Mean Total serum cholesterol (mmol/L)	4.3±1.2	4.1±1.1	p** = **0.097
At least 150 mins moderate intensity exercise per week (%)^§^	58 (36.7%)	95 (55.9%)	**p = 0.001**
“High risk” alcohol intake (%)^§ ¶^	9 (5.9%)	41 (25.6%)	**p<0.001**
“Poor” sleep quality (%)	51 (32.3%)	37 (22.0%)	**p = 0.037**
Current cigarette smoker (%)^§^	12 (7.6%)	32 (18.8%)	**p = 0.003**
**Co-morbidity profile**			
Hypertension (%)	123 (76.4%)	117 (67.2%)	p** = **0.063
CAD (%)	40 (24.8%)	72 (41.4%)	**p = 0.001**
Type 2 diabetes (%)	45 (28.0%)	51 (29.3%)	p** = **0.783
Stroke/Systemic Embolism/TIA (%)	21 (13.0%)	31 (17.8%)	p** = **0.228
COPD (%)	19 (11.8%)	35 (20.1%)	**p = 0.039**
Thyroid dysfunction (%)	18 (11.2%)	8 (4.6%)	**p = 0.024**
Depression (%)	56 (34.8%)	40 (23.0%)	**p = 0.017**
Renal Impairment (eGFR<60 mL/min/1.73 m^2^, %)	70 (43.8%)	47 (27.2%)	**p = 0.002**
Obesity (BMI ≥30 kg/m^2^, %)^≠^	69 (49.6%)	59 (37.3%)	**p = 0.033**
Mean LVEF (%)	61.4%±10.3%	54.4%±13.0%	**p = 0.005**
Mean MoCA score	23±4	23±4	p** = **0.828
Mean SF-12 HRQoL physical component score (PCS)	34.7±11.5	40.6±11.5	**p<0.001**
Mean SF-12 HRQoL mental component score (MCS)	49.3±11.9	50.7±10.8	p** = **0.264
Mean EQ-5D HRQoL score	0.716±0.202	0.759±0.244	p** = **0.084
**Treatment profile**			
Beta Blocker (%)	77 (47.8%)	88 (50.6%)	p** = **0.615
Digoxin (%)	64 (39.8%)	53 (30.5%)	p** = **0.075
Anti-arrhythmic (%)	51 (31.7%)	50 (28.7%)	p** = **0.558
Diuretic (%)	84 (52.2%)	55 (31.6%)	**p<0.001**
Warfarin (%)	87 (54.0%)	99 (56.9%)	p** = **0.599
Aspirin only (%)	75 (46.6%)	86 (49.4%)	p** = **0.603
Aspirin plus Clopidogrel (%)	29 (18.0%)	33 (19.0%)	p** = **0.822
Premature discontinuation of anti-thrombotic therapy (%)	40 (24.8%)	45 (25.9%)	p** = **0.831

* Assessed in n** = **325 patients; ^†^ Assessed in n** = **276 patients; ^≠^ Assessed in n** = **297 patients; ^§^ Assessed in n** = **328 patients; ^¶^ defined as consumption of >2 standard drinks on any occasion (on average).

AF  =  Atrial Fibrillation; CHA_2_DS_2_-VASc score definition: C  =  *congestive heart failure/LV dysfunction* (1 point), H  =  *hypertension* (even if treated; 1 point), A_2_ **=** *age* ≥*75 years* (2 points), D** =** *diabetes mellitus* (1 point), S_2_ **=** *stroke/SE/TIA* (2 points), V** =** *vascular disease* (1 point), A** =** *age 65*–*74 years* (1 point), Sc  =  *sex category* (female sex; 1 point); HR  =  Heart Rate; bpm  =  beats per minute; SBP  =  Systolic Blood Pressure; DBP  =  Diastolic Blood Pressure; BMI  =  Body Mass Index; HDL  =  High Density Lipoprotein; LDL  =  Low Density Lipoprotein; CAD  =  Coronary Artery Disease; PVD  =  Peripheral Vascular Disease; TIA  =  Transient Ischaemic Attack; COPD  =  Chronic Obstructive Pulmonary Disease; eGFR  =  estimated Glomerular Filtration Rate; LVEF  =  Left Ventricular Ejection Fraction; MoCA  =  Montreal Cognitive Assessment; SF-12  =  Short Form 12 items questionnaire; HRQoL  =  Health-Related Quality of Life; PCS  =  Physical Composite Score; MCS  =  Mental Composite Score; EQ-5D  =  EuroQol Five-Dimensional questionnaire; INR  =  International Normalised Ratio.

### AF antecedents

As shown in **Table 1**, women demonstrated a higher mean body mass index (BMI) than men reflecting that, on average, women were classified as “obese” and men as “overweight”. Just over one third of women undertook the recommended levels (specifically for older individuals) of exercise in Australia [25] compared to over half of their male counterparts. Only a minority of women (6%) consumed alcohol at “high risk” levels (considered more than two standard drinks on any one occasion [26]), compared to over a quarter of male patients. A similar proportion of women reported currently smoking cigarettes. Over double the proportion of men compared to women reported current cigarette smoking. Self-reported average sleep quality was “poor” according to one third of the female patients; however, the same reports were made by 22% of their male counterparts (p** = **0.037).

### AF-specific profile

Women and men showed a similar pattern of basic AF characteristics (**Table 1**). Future thrombo-embolic risk, as calculated via the CHA_2_DS_2_-VASc score was significantly higher in women than men.

### Co-morbidity profile

Gender-based differences in the co-morbidity of the study cohort were also evident (**Table 1** **)**. Both concomitant coronary artery disease (CAD) and chronic obstructive pulmonary disease (COPD) was more common in men (41.4% versus 24.8% and 20.1% versus 11.2%, respectively). Conversely, it was significantly more common for women to present with co-morbid thyroid dysfunction (previously ruled out as the cause of AF), depression, renal impairment and obesity. Women had more preserved cardiac function as reflected by a higher mean LVEF than men (61.4%±10.3% versus 54.4%±13.0%; p** = **0.005). With respect to HRQoL, the only difference was that women had more overall physical limitations (as determined by a lower composite score of the SF-12; p<0.001).

### Treatment profile

Nominated rate or rhythm control of underlying AF was similar for men and women (approximately 64% for rate and 36% for rhythm, p** = **0.847 for both) and this was reflected in the most commonly prescribed medications (beta blocker, digoxin or anti-arrhythmic) to achieve therapeutic goals in AF. Despite increased thrombo-embolic risk in women, there were no discernible differences in anti-thrombotic use (warfarin, aspirin alone or in combination with clopidogrel) between the sexes. When anti-thrombotic therapy was further assessed, approximately 25% of both sexes reported premature and permanent treatment discontinuation due to, most commonly, a procedure requiring therapy withdrawal (7.5% versus 12.1% for women versus men; p** = **0.157), their physician’s decision (6.2% versus 5.7%; p** = **0.858) or a bleeding episode (6.8% versus 4.0%; p** = **0.255). Finally, it was significantly more common for women to be prescribed diuretic therapy than for men (52.2% versus 31.6%; p<0.001).

### Symptom and trigger profile

There were gender-related differences present in AF symptoms self-reported by patients. Of the more typical symptoms commonly described by patients relating to the presence of AF (or rapid AF for those with permanent AF), significantly more women than men reported fatigue or lethargy (56.5% versus 44.3%; p** = **0.025), palpitations or “fluttering” (63.4% versus 38.5%; p<0.001) and weakness (43.5% versus 29.9%; p** = **0.010). Alternatively, no gender-based differences were observed in respect to self-reported triggers of AF (stress, overexertion, illness, medications or alcohol).

### Age-adjusted comparisons of women and men

**Figure 2:**
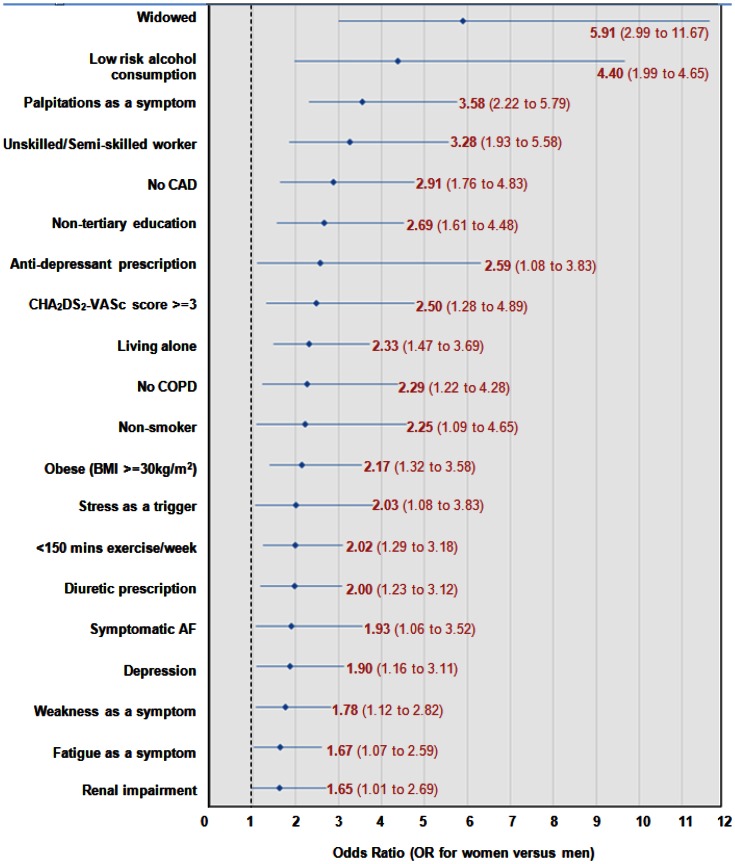
Age-adjusted differences in the profile of women (versus men) recruited into SAFETY.

**Figure 2** provides a summary of age-adjusted comparisons of the demographic and clinical characteristics of women and men. From a demographic perspective, women were more than twice as likely to live alone and were nearly six times more likely to be a widow. In addition, women were almost three times more likely to have non-formal tertiary education and to have worked in an unskilled/semi-skilled occupation (i.e. clerical/ administration/ community worker/ personal/ customer service/ labourer). Clinically, women were approximately twice as likely to experience symptomatic AF (particularly palpitations, weakness and fatigue), have a high thrombo-embolic risk (indicated by a CHA_2_DS_2_-VASc score of 3 or more), be classified as obese and have renal impairment, but no medical history of CAD or COPD. Furthermore, stress was the most likely trigger of an episode of AF (or rapid AF) in double the proportion of women to men. With regard to lifestyle factors, women were almost 4.5 times more likely to consume alcohol at “low risk” levels, twice as likely to be a non-smoker but unlikely to undertake the recommended weekly levels of exercise. From a treatment perspective, women were about 2.5 times more likely to be prescribed an anti-depressant than men and twice as likely to be receiving diuretic therapy.

## Discussion

**Figure 3:**
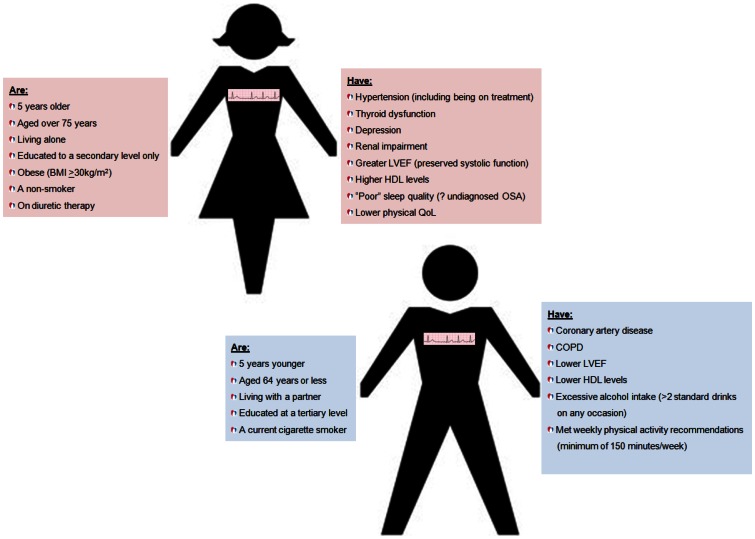
Typical socio-demographic and risk profiles of women versus men with chronic AF in the SAFETY cohort.

Given parallel issues relating to high levels of morbidity and mortality, there remains a paucity of studies examining the potential impact of AF-specific management programs with the potential to deliver equivalent improvements in health outcomes similar to those applied to patients with CHF [4]. Like the seminal trials of CHF management programs, there is the potential to address critical gender imbalances in developing evidence-based therapeutics; via the same pattern of recruiting older, more complex and more gender-balanced study cohorts who more accurately reflect real-world patient populations. With the notable exception of the earliest report of AF-specific management from our group [27], and the largest AF-specific management trial to date (involving 712 patients) [28], SAFETY represents one of the largest studies of its kind. In this detailed examination of the SAFETY cohort from a gender-specific perspective, we can not only confirm a more even balance of men and women consistent with the broad epidemiology of AF that contrasts with key clinical trials, but some important differences that will likely influence individualised management. As represented by **Figure 3**, the “typical” demographic and clinical profile of female and male patients in the SAFETY cohort (and by inference the wider AF patient population) is markedly different. Of particular interest, in our cohort, typically older women were more socially isolated and less educated. While this is not surprising given the social history of Australia (as with many other countries), these factors have potentially important implications for disease-specific knowledge, symptom recognition and seeking medical assistance. Furthermore, we have recently highlighted the critical importance of mild cognitive impairment in older individuals with AF, recommending systematic screening to determine those at risk and/or requiring modification to any educational programs, although this applies equally to women and men [29].

Our findings are consistent with those of other studies reporting that women with AF were older, had a higher symptom burden, had poorer HRQoL, were more likely to be treated with diuretics, had an increased prevalence of hypertension, and that men with AF had a higher prevalence of CAD [14]–[17].The Symptom Mitigation in Atrial fibRillaTion (SMART) Study by Goli et al. showed that patients with severe AF symptoms were more likely to be female and less educated [30]. The current study provides an extension to previous data by identifying additional socio-demographic, lifestyle, clinical and therapeutic factors of potential importance to longer-term health outcomes and individualised management. When considering AF-specific management studies, some potentially influential differences between the cohorts exist. Age profiles of the nurse-led care and usual care cohorts of the AF-Clinic study conducted by Hendriks et al. comprising 44.7% and 37.9% females, were 66±13 years and 67±12 years, respectively [28]. Female and male patients in the current study were 7–8 years and 2–3 years older than participants of AF-Clinic, respectively. Furthermore, patients of the current research were high-risk, hospitalised patients unlike the healthier participants recruited by Hendriks et al. within primary care clinics [28].

Common identification of gender-related diversity may potentially reflect different pathways of development and/or promotion of AF between the sexes. From the current data, it appears that the pathway to AF in men is related to CAD and/or ischaemic heart disease. However, for women, AF was more commonly associated with co-morbid conditions (hypertension, obesity, thyroid and renal dysfunction). It is also possible that women experience AF differently to their male counterparts. Historically, oestrogen was implicated as the cause of the gender-related diversity in CVD and extensive observational data supported this theory. However, recent prospective clinical trials have shown that oestrogen therapy alone or in combination with progestin does not protect from CVD or stroke and may even be harmful [31], [32]. Electrophysiologic differences throughout the atrial tissue (e.g. in the atrial effective refractory period) and hormonal differences are now suggested as potential mechanisms [6]. More broadly, differences in serum cholesterol, BMI and diabetes prevalence being responsible for approximately 50% of the gender-related disparity between women and men with CVD have also been described [33]; a similar relationship could be assumed for AF. The prescription of evidence-based treatment strategies may also be influenced by the gender of the presenting individual [6]. Here, we have described the influence of gender on gold-standard anti-thrombotic AF-treatment; women are at higher thrombo-embolic risk, yet treatment regimens remain no different to those of men. Further, women experience a greater symptom burden, indicating that standards for rate (or rhythm) control may also show gender inequality. It is also possible that the involved therapeutic regimens required for AF management may be too complex to be optimised for typically less educated women with a small (if existent) support network.

The differences identified in the current research have important clinical implications for the management of patients with AF based on their gender. Utilising more specific factors to formulate and deliver individualised disease management programs may enable improvement of clinical outcomes. A higher symptom burden identified in women suggests the need for increased education into symptom recognition and clinical impact, ensuring that help is sought as early as possible before major deterioration. To this end, design and delivery of educational programs targeted at assisting patients to develop an inventory of skills relating to when to seek medical attention is essential. Women are at greater risk of AF-related complications due to being older at presentation, more clinically complex and at greater thrombo-embolic risk than their male counterparts within the context of similar prescription and delivery of AF-specific therapeutic regimens. Difficulty in interpreting the severity of symptoms of acute coronary syndrome was shown to be most predictive in female patients who delay seeking care [34]. Confusion in symptom identification, interpretation and perception and their vulnerability to heart disease by women and health care professionals has led to a delay in seeking care for cardiac problems that can reduce the success of treatment or prevent the use of some interventions [35]. Increasing knowledge of different patients’ experiences of AF would allow for education to be adjusted accordingly with the ultimate aim of preventing readmissions and longer-term morbidity and mortality.

Awareness of specific gender-related differences is critical when applying disease management programs that include examining the effectiveness of newly developed therapeutics for the medical management of chronic diseases. As clinical trials inform the advancement of clinical practice, it is useful to understand the ready application of results from recent positive trials in patients with AF to patients in “real world” clinical practice. Comparison of “real world” AF patients with clinical trial cohorts shows that clinical differences may have implications for balancing benefit and risk when applying standardised therapies to high risk AF patients. Differing from recent clinical trial cohorts, SAFETY is most reflective of the true ratio of high risk women to men with AF who present to hospital (see **Table 2**). It is likely that gender-based differences in AF will be exacerbated in the next decade or more; for example, in Australia it is predicted that there will be equal numbers of females aged <5 years as those >80 years (far outnumbering equivalent aged men). Much research is still required to complete the picture of the physiology behind gender-specific differences in patients with AF; the current research contributes somewhat to its elucidation. It will also be important to learn if these differences extend beyond hospitalisation. Confirmation of gender-specific differences in patients with chronic AF highlights a need to identify and accommodate differences into disease management programs. Otherwise, clinical outcomes may be compromised. Awareness of key gender-specific differences in AF allows for formulation and delivery of a safer, more effective and individualised approach to clinical management with patient stratification into well-known risk groups.

**Table 2:** Proportion of women represented in recent AF clinical trials versus the SAFETY cohort.

Clinical Trial	Year of publication	Number of female participants (n; %)	Mean age of female participants (yrs±SD)	Number of male participants (n; %)	Mean age of male participants (yrs±SD)	Mean/median age of all participants (yrs±SD/[IQR])
**SAFETY**	**2013**	**n = 161 (48.1%)**	**74.0±10.3**	**n = 174 (51.9%)**	**69.3±11.6**	–
ARISTOTLE	2011	n** = **6,416 (35.3%)	–	n** = **11,785 (64.7%)	–	70 (63 – 76)
AVERROES	2011	n** = **2,322 (41.5%)	–	n** = **3,277 (58.5%)	–	70.0±10.0
ROCKET-AF	2011	n** = **5, 663 (39.7%)	–	n** = **8,601 (60.3%)	–	73 (65 – 78)
RACE II	2010	n** = **211 (34.4%)	–	n** = **403 (65.6%)	–	68.0±8.0
RE-LY	2009	n** = **6,599 (36.4%)	–	n** = **11,514 (63.6%)	–	71.5±8.7
AFFIRM	2002	n** = **1,594 (39.3%)	–	n** = **2,466 (60.7%)	–	69.7±9.0
RACE	2002	n** = **191 (36.6%)	–	n** = **331 (63.4%)	–	68.0±8.0

SAFETY  =  Standard versus Atrial Fibrillation spEcific managemenT study.

ARISTOTLE  =  Apixaban for Reduction in Stroke and Other Thromboembolic Events in Atrial Fibrillation Study.

AVERROES  =  Apixaban Versus Acetylsalicylic Acid to Prevent Stroke in Atrial Fibrillation Patients Who Have Failed or Are Unsuitable for Vitamin K Antagonist Treatment.

ROCKET-AF  =  Rivaroxaban Once daily oral direct factor Xa inhibition Compared with vitamin K antagonism for prevention of stroke and Embolism Trial in Atrial Fibrillation).

RACE  =  RAte Control versus Electrical Cardioversion for Persistent Atrial Fibrillation Study.

RE-LY  =  Randomised Evaluation of Long term anticoagulant therapy.

AFFIRM  =  Atrial Fibrillation Follow-up Investigation of Rhythm Management.

There are a number of limitations that require comment. Firstly, the respective cohorts of women and men analysed are relatively small and focused only on those who are high risk and have been hospitalised in Australia (with its hybrid universal health/private health care system). Therefore, findings from the current research may not be readily applicable to all women and men with AF in different countries and health care settings. Further, the cohort consists of those who are acutely ill, potentially exaggerating clinical stability and affecting generalisability of the findings. Exclusion of non-English speaking individuals into the study may have introduced selection bias, although English competency is necessary for comprehensive understanding of the many questionnaires administered. Furthermore, the often chaotic acute clinical setting under which the initial comprehensive assessment was conducted in addition to the patients’ altered health state may not represent an ideal testing situation. Despite the selection of a “real world” cohort, we cannot discount the possibility of bias in study selection (perhaps still under-representing females despite screening data) and these data may not readily apply to other hospitalised cohorts.

Whether observed differences in this cohort (particularly in respect to treatment) translate to differential health outcomes is still unknown (study follow-up will be complete in late 2013). Despite these limitations, these data have important clinical implications for the post-discharge management of patients with chronic AF.

## Conclusion

Gender differences identified in high risk individuals hospitalised with chronic AF must be recognised as valid stratifying features for the treatment and prevention of poorer health outcomes in this patient group. Health care providers should be cognisant of these defining features in an attempt to individualise and, hence, optimise the management of patients with chronic AF and reduce potential morbidity and mortality.
